# A Cross-Sectional Survey of Dentists on Antibiotic Prescribing and Allergy Management in Oral Surgery

**DOI:** 10.3290/j.ohpd.c_2391

**Published:** 2025-12-16

**Authors:** Paniz Golchini, Sayna Behkar, Ömer Faruk Kocamaz, Serpil Altundoğan

**Affiliations:** a Paniz Golchini PhD Student, Department of Orthodontics, Gülhane Faculty of Dentistry, University of Health Sciences, Ankara, Turkey. Conceptualisation of the study, formulation of the hypothesis, study design, preparation of figures and tables, and manuscript writing.; b Sayna Behkar PhD Student, Department of Periodontology, Gülhane Faculty of Dentistry, University of Health Sciences, Ankara, Turkey. Data collection, literature review, execution of experiments, and contribution to manuscript drafting.; c Ömer Faruk Kocamaz Research Assistant, Department of Oral and Maxillofacial Surgery, Ankara University Faculty of Dentistry, Ankara, Turkey. Interpretation of results, contribution to the discussion section, final review and editing.; d Serpil Altundoğan Professor, Department of Oral and Maxillofacial Surgery, Ankara University Faculty of Dentistry, Ankara, Turkey. Supervision of data collection, data verification, and proofreading.

**Keywords:** antibacterial agents, antimicrobial stewardship, dentists, drug hypersensitivity, oral surgical procedures

## Abstract

**Purpose:**

This study aimed to evaluate antibiotic prescribing habits, guideline awareness, and allergy management practices among dentists performing oral surgical procedures in Turkey.

**Materials and Methods:**

A descriptive cross-sectional survey was conducted with 263 dentists between January and March 2025. A 25-item questionnaire collected data on demographics, prescribing criteria, prophylactic use, guideline adherence, allergy/hypersensitivity management, and educational needs. Ethical approval was obtained from the Ankara University Faculty of Dentistry Ethics Committee (Decision No: 7/1). The data were analysed via descriptive statistics and Chi-square tests (P <0.05).

**Results:**

Penicillin-based antibiotics were most preferred (95.4%). Only 18.3% of the patients consistently followed clinical guidelines, and 16.0% referred patients for allergy testing. While 58.6% chose alternative antibiotics in suspected allergy cases, only 35.4% always informed patients about potential side effects. Awareness of national antibiotic guidelines was reported by 69.6%, but only 33.1% applied them. Statistically significant associations were found between professional title and both guideline adherence (P = 0.015) and monthly prescribing frequency (P = 0.018). Most dentists (77.9%) disagreed with stopping antibiotics when symptoms improved, preferring full courses.

**Conclusion:**

Dentists in Türkiye frequently rely on empirical antibiotic prescribing in oral surgical procedures, with limited adherence to available clinical guidelines and insufficient attention to allergy management. Although awareness of antimicrobial resistance is relatively high, its translation into evidence-based practice remains inadequate. These findings emphasise the need for clearer national protocols, incorporation of antibiotic stewardship into dental education, and enhanced clinical training in allergy recognition to promote safer and more rational antibiotic use.

The use of antibiotics is widespread in dental practice, particularly in oral surgical procedures such as tooth extractions, implant placements, and apical surgeries. They are essential for preventing perioperative infections in medically compromised patients.^5,12^ Dentists frequently prescribe antibiotics for both therapeutic and prophylactic purposes, making them key actors in antimicrobial stewardship.^4^ However, overuse and inappropriate prescriptions remain common, contributing significantly to the global health problem of antimicrobial resistance (AMR).^15,22^

Recent evidence shows that dentists significantly contribute to antibiotic overuse by prescribing antibiotics when not clinically indicated or by unnecessarily extending treatment durations.^20,21^ In oral surgical settings, prescriptions are often based on individual preferences rather than evidence-based guidelines,^13^ with antibiotics sometimes given ‘just in case’. Such practices increase the risk of adverse outcomes and further drive AMR.

An important but often overlooked consequence of unnecessary antibiotic use is allergic and hypersensitivity reactions, ranging from mild gastric irritation or rashes to life-threatening anaphylaxis.^9,14,19^ Failure to recognise or manage such reactions can result in severe patient harm. However, studies have shown that many dentists feel inadequately prepared for allergy recognition and management in daily practice.^3,8^

Although international evidence-based guidelines exist to support appropriate antibiotic use in oral surgery, a clear gap remains between these recommendations and actual clinical practice.^11,27^ This discrepancy highlights the need to evaluate dentists’ prescribing behaviours to identify areas for improvement.

Many dentists still rely on personal experience or outdated knowledge rather than standardised recommendations, leading to inconsistent practices.^1^ In Turkey, several surveys have assessed dentists’ general attitudes toward antibiotic use, but few have specifically examined prescribing criteria in oral surgery, commonly used antibiotics, or approaches to allergy management.^28^

This study explores Turkish dentists’ views and practices regarding antibiotic use in oral surgical procedures, with a particular focus on the management of hypersensitivity reactions and antibiotic-related allergies – an area rarely addressed in the literature. A structured questionnaire was used to assess antibiotic selection, duration of use, guideline adherence, frequency and management of allergic reactions, and access to educational resources. The findings provide insight into prescribing dynamics, highlight areas requiring further training and stricter protocol compliance, and support the development of improved educational initiatives to guide clinical decision-making in dental practice.

## MATERIALS AND METHODS

This cross-sectional and descriptive study was conducted to evaluate dentists’ practices, attitudes, and awareness regarding antibiotic prescribing, allergy management, and hypersensitivity reactions in oral surgical procedures in Turkey. The Ankara University Faculty of Dentistry ethics committee granted ethical approval prior to the start of the study (Approval No: 7/1, Date: 10.03.2025). A written consent statement at the start of the questionnaire was used to obtain informed consent from each participant. The guiding principles of the Declaration of Helsinki were followed when the study was conducted.

A structured questionnaire with 25 closed-ended questions was used as the data collection technique. It was created by the study team on the basis of clinical knowledge and thorough literature analysis. The questionnaire covered topics such as demographics, antibiotic prescribing criteria, prophylactic use habits, adverse effects of antibiotics, allergy and hypersensitivity management, clinical guideline usage, access to educational resources, and awareness of antibiotic resistance. The full version of the questionnaire used in this study is provided in English as a supplementary file.

A pilot study was carried out to evaluate the questionnaire items’ applicability, clarity, and comprehensibility prior to the main data collection process. Forty dentists with a variety of clinical and academic backgrounds participated in the pilot phase. Feedback on the questions’ phrasing, applicability, and structure was requested from the participants. One item was changed to eliminate ambiguity and increase clarity on the basis of their answers. The questionnaire was generally well understood, according to the pilot study, and no significant structural adjustments were thought to be needed. The final analysis did not include the pilot data.

A power calculation was performed in advance to determine the required number of participants for the study. On the basis of a two-tailed binomial test with an assumed effect size (g) of 0.10, a significance level of 0.05, and a statistical power of 0.90 (1–β), the sample size required was calculated to be 263 participants. This sample size ensures that the study will be adequately powered to detect modest but significant differences between dentists’ awareness and practices. Power analysis was performed via G*Power software.

Dentists were eligible to participate in the study if they were currently practising in a private clinic, public hospital, or university hospital; had never taken part in a similar study; voluntarily agreed to participate; had no diagnosed psychological disorders; and were willing to complete the entire questionnaire. Conversely, candidates were excluded if they had previously participated in a similar study, declined to participate voluntarily, were known to have a severe psychological disorder, refused to complete the questionnaire or provided incomplete responses.

Data were collected during the first quarter of 2025 via a face‒to‒face survey method. Participation was voluntary, and 17 dentists declined to complete the questionnaire. A total of 263 of the 280 people who were contacted finished the survey, yielding a response rate of approximately 93.9%. Every response was gathered anonymously, and the participants’ personal information was kept completely private.

To minimise interviewer bias, every researcher applied a standardised protocol for describing the study and the questionnaire during face‒to-face interviews. The study purpose and confidentiality were communicated to the participants. Each participant signed and read a written informed consent form prior to responding to the survey. This provided voluntary and independent participation and enhanced ethical rigour and data reliability.

A step-by-step flowchart was created to outline the methodological process of this study. It includes the development and design of the questionnaire, the evaluation of question reliability, the administration of the final version, and subsequent data analysis via SPSS version 21.0. Visual representation facilitates a clear understanding of the research progression from initial planning to result interpretation.

**Fig 1:**
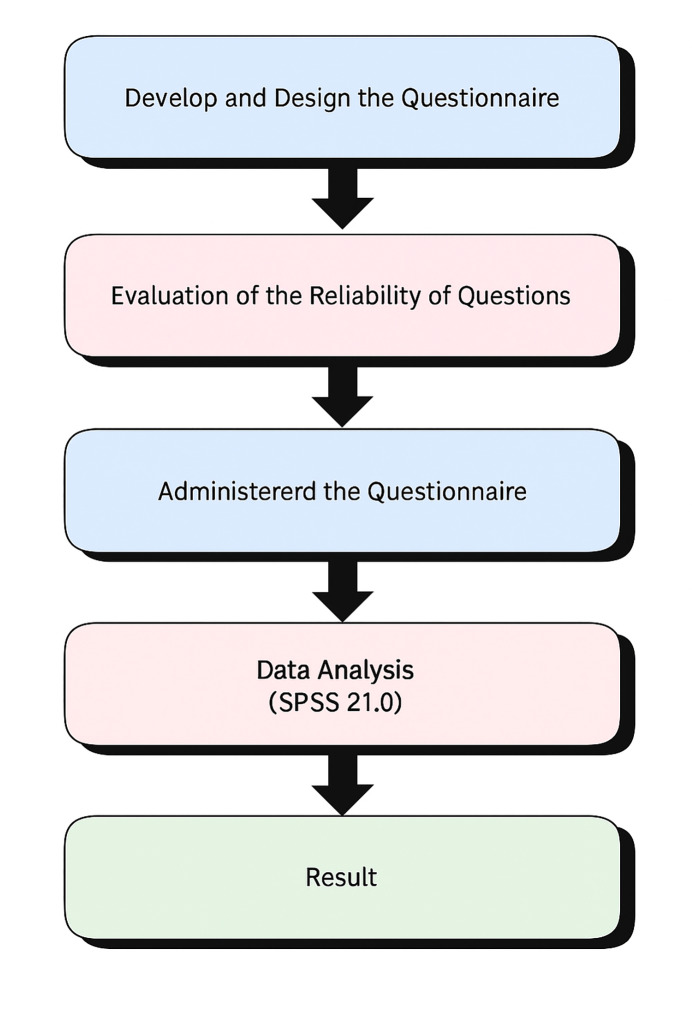
Flowchart representing the methodological process of the study, including questionnaire development, reliability assessment, data collection, and analysis.

The detailed process is illustrated in Figure 1.

### Data Analysis

The data collected were analysed via IBM SPSS Statistics 21.0 (BM, Armonk, NY, USA). Survey results and participant information were compiled via descriptive statistics, such as frequencies and percentages. The Chi-square (χ^2^) test was used to examine associations between categorical variables. In cases where the expected cell frequencies were less than 5, Monte Carlo simulation was applied to support the P values. A P value of <0.05 was considered statistically significant.

## RESULTS

### Demographic Characteristics

The final sample consisted of 263 dentists. Among them, 55.9% (n = 147) were women, and 44.1% (n = 116) were men. The most dominant age group was 20–30 years (65.0%, n = 171), followed by equal numbers in the 31–40 and 41–50 age groups (11.4% each), and 12.2% (n = 32) were aged 51 and older.

For professional experience, 58.9% (n = 155) practised 0–5 years, and 15.6% (n = 41) had 16 or more years of experience. Most respondents were PhD students or research assistants (60.8%, n = 160), with fewer numbers of specialist dentists (16.7%), university faculty (13.3%), and general dentists (9.1%).

Most participants were employed at public universities (58.9%, n = 155), 39.5% (n = 104) were employed in private clinics, and only 1.5% (n = 4) were employed in public hospitals.

Regarding specialities, oral and maxillofacial surgery (22.1%), periodontology (18.3%), and prosthodontics (12.9%) were the most represented specialities (Table 1).

**Table 1:** Demographic data of the participants

Demographic data	Number (n)	Percentage (%)
Age	20–30 years old	171	65.0
31–40 years old	30	11.4
41–50 years old	30	11.4
51 years old ≤	32	12.2
Total	263	100.0
Gender	Female	147	55.9
Male	116	44.1
Total	263	100.0
Working Sector	Private sector	104	39.5
Public hospital	4	1.5
Public university	155	58.9
Total	263	100.0
Working experience	0–5 years	155	58.9
6–10 years	36	13.7
11–15 years	31	11.8
16 years and above	41	15.6
Total	263	100.0
Title	General dentist	24	9.1
Specialist dentist	44	16.7
University professor	35	13.3
Research assistant/PhD student	160	60.8
Total	263	100.0
Specialty	Orthodontics	16	6.1
Pedodontics	21	8.0
Endodontics	33	12.5
Periodontology	48	18.3
Oral and maxillofacial surgery	58	22.1
Oral diagnosis and radiology	18	6.8
Prosthodontics	34	12.9
Restorative dentistry	12	4.6
None	23	8.7
Total	263	100.0


### Questionnaire Results and Sectional Findings

For antibiotic prescribing practices, 52.9% (n = 139) of the people who took part in the study reported that the most important factor for the prescription of antibiotics in oral surgical procedures was the infection risk level. Only 18.3% (n = 48) of the participants used clinical guidelines as their only reference, and only 14.8% (n = 39) referred to the patient’s general health.

When asked to identify their most preferred antibiotics, an overwhelming majority of 95.4% (n = 251) reported the use of penicillin. Other alternatives, such as metronidazole (2.3%), clindamycin (1.5%), and azithromycin (0.8%), were less commonly selected.

**Fig 2:**
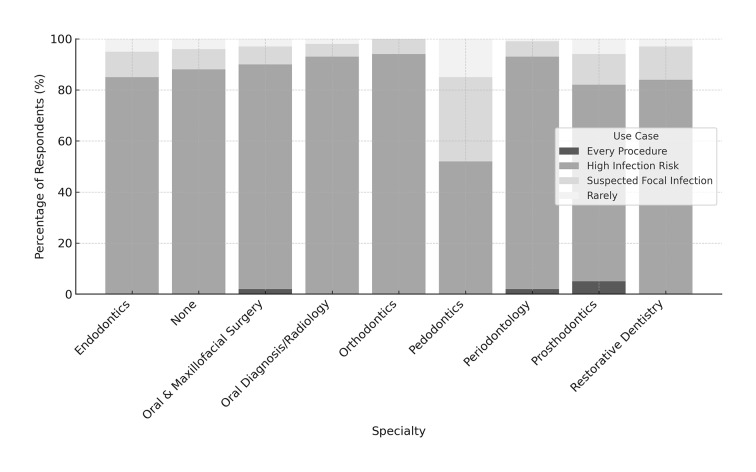
Prophylactic antibiotic use preferences by speciality, showing distribution across every procedure, high infection risk, suspected focal infection, and rare use.

According to the results, most dentists across all specialities reported prescribing prophylactic antibiotics, mainly in cases of high infection risk. This was especially common in oral and maxillofacial surgery (95%), periodontology (90%), oral diagnosis/radiology (89%), endodontics (88%), and general practitioners who selected ‘None’ (87%). In orthodontics and prosthodontics, this rate was slightly lower, approximately 80%, and lowest in pedodontics (60%). The second most common reason was suspected focal infection, particularly among pedodontists (25%) and orthodontists (15%). A small number reported using antibiotics for every procedure, especially prosthodontics (5%) and oral surgery (1%). Only restorative dentistry stood out, with 100% of respondents stating that they rarely prescribe antibiotics, which is consistent with the low infection risk in that field (Fig 2).

**Fig 3:**
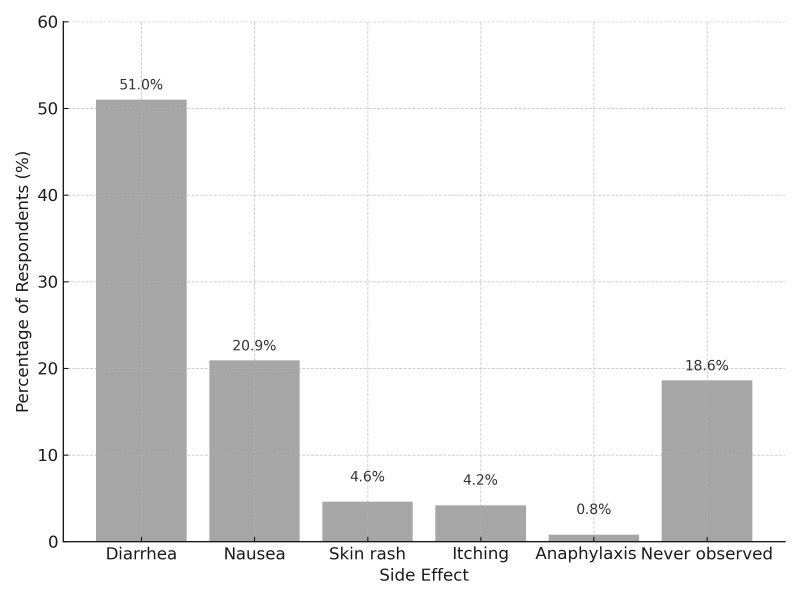
Most common side effects after antibiotic use.

Antibiotic prescribing protocols were mostly based on international guidelines (75.7%) (n = 199), whereas only 11.0% (n = 29) followed national guidelines. Notably, as Türkiye currently lacks unified dental-specific national antibiotic guidelines, the term ‘national guidelines’ in the questionnaire may reflect broader national frameworks or institutional protocols. Side effects associated with antibiotics were common: diarrhoea (51.0%) (n = 134) was the most frequently reported side effect, followed by nausea (20.9%) (n = 55) (Fig 3).

**Fig 4:**
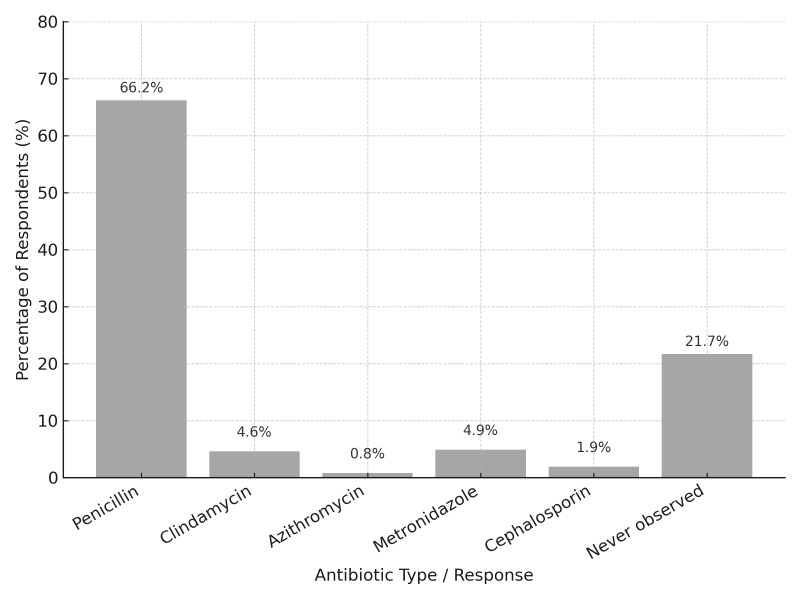
Antibiotics are most commonly associated with allergic reactions.

Penicillin was also the antibiotic most strongly associated with allergic reactions (66.2%) (n = 174) (Fig 4).

For the sake of allergy control, 58.6% (n = 154) of the respondents said they would prefer to prescribe another antibiotic, but only 16.0% (n = 42) referred patients to a specialist for testing allergies. When explaining side effects to patients, just 35.4% (n = 93) said they always did so, whereas the majority (57.4%, n = 151) sometimes did. Knowledge of national antibiotic guidelines was limited: only 33.1% (n = 87) of respondents were both aware of and applying them, whereas 30.4% (n = 80) lacked any knowledge of these guidelines. The frequency of hypersensitivity reactions was ‘rare’ (54.0%, n = 142), while 23.2% (n = 61) reported never having encountered a case.

When asked whether they take sufficient precautions to minimise side effects, only 34.6% (n = 91) agreed or strongly agreed. Moreover, 77.9% (n = 205) of the participants disagreed with the cessation of antibiotics when their symptoms improved, reflecting support for completing the full course of antibiotics. For the management of allergies, 82.9% (n = 218) of the subjects had an employed patient history, whereas 14.1% (n = 37) had objective allergy testing.

Most participants reported that their knowledge of the consequences of excessive use of antibiotics was good (55.5%) (n = 146) or very good (17.1%) (n = 45). However, only 39.5% (n = 104) of the participants believed that they had been sufficiently educated in the management of allergies. Furthermore, 52.4% (n = 138) of the participants believe that Turkish dentists are not well educated regarding antibiotic allergies and hypersensitivity reactions. When asked what the best way is to access education related to antibiotic use and allergy management, the respondents voiced a clear preference for evidence-based and self-access materials. The highest voted option was reading scientific literature (47.5%, n = 125), followed by online courses (26.2%, n = 69) and in-service training sessions organised at their facilities (17.9%, n = 47). Very few (8.4%, n = 22) of them voted for conferences as their first-choice educational venue. These findings show the participants’ preference for flexible, current, and academically oriented learning formats.

**Fig 5:**
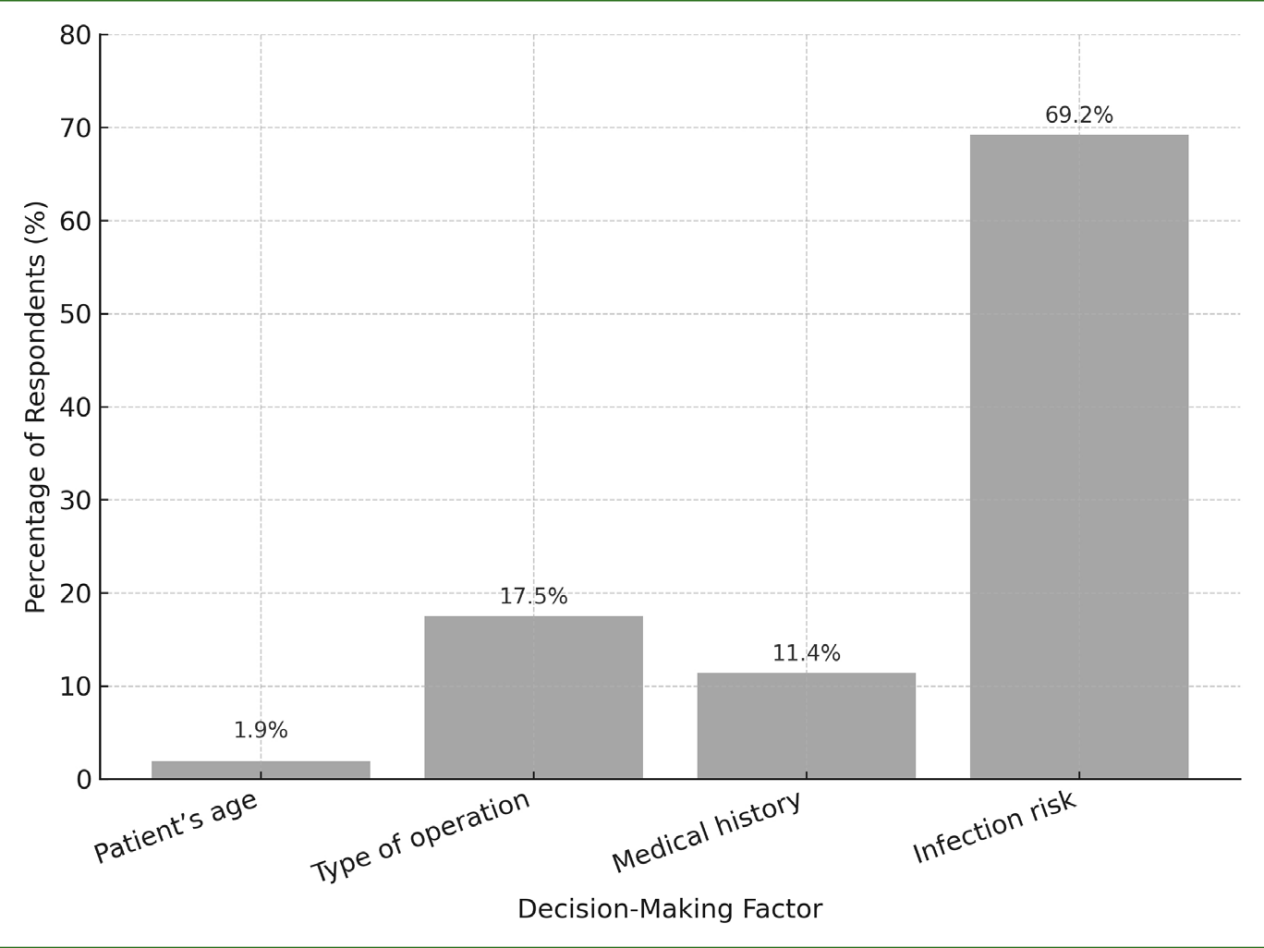
Primary factors influencing postoperative surgery antibiotic prescription, with infection risk being the most common, followed by type of operation, medical history, and patient age.

When determining the main criterion for postoperative antibiotic application, 69.2% (n = 182) used infection risk as the deciding factor, whereas only 1.9% (n = 5) considered patient age important (Fig 5).

Notably, 62.4% (n = 164) of the participants considered national antibiotic guidelines to be too lacking, and only 1.9% (n = 5) were extremely sufficient. In terms of clinical practice patterns, 44.1% (n = 116) of the participants prescribed antibiotics in 0–10% of their monthly oral surgery cases, whereas 13.3% (n = 35) did so in more than half of their cases. Over the past year, 44.9% (n = 118) had to change their treatment protocols for 1–5 patients due to side effects.

Chi-square tests were used to explore associations between significant demographic variables and antibiotic prescribing behaviour:

A significant association was observed between the respondents’ professional titles and their awareness and adherence to national guidelines on antibiotic use in oral surgical procedures (x^2^ = 15.9, P = <0.05). Notably, research assistants and specialist dentists demonstrated higher levels of awareness and compliance than general practitioners did.

Differences in the frequency of monthly antibiotic prescriptions were also statistically significant across professional titles (x^2^ = 18.1, P = <0.05). In general, dentists reported prescribing antibiotics in a greater proportion of oral surgery cases than PhD students and academics did (Table 2).

**Table 2:** Awareness of antibiotic guidelines and frequency of prescriptions by professional title

	Title	Chi-square analysis
General dentist	Specialist dentist	University professor	Research assistant/PhD Student	Total
n (%)	n (%)	n (%)	n (%)	n (%)	Chi-square	P
Awareness of the latest guidelines	Yes, I am aware and I follow them	3(12.5)	15 (34.1)	10 (28.6)	59 (36.9)	87 (33.1)	15.9	0.015
I am aware, but I do not always follow them	17 (70.8)	16 (36.4)	15 (42.9)	48 (30.0)	96 (36.5)
I am not aware of the guidelines	4 (16.7)	13 (29.5)	10 (28.6)	53 (33.1)	80 (30.4)
Total	24 (100.0)	44 (100.0)	35 (100.0)	160 (100.0)	263 (100.0)
Frequency of monthly antibiotic prescription	0–10%	5 (20.8)	22 (50.0)	22 (62.9)	67 (41.9)	116 (44.1)	18.1	0.018
11–30%	5 (20.8)	11 (25.0)	3 (8.6)	37 (23.1)	56 (21.3)
31–50%	11 (45.8)	6 (13.6)	6 (17.1)	33 (20.6)	56 (21.3)
51% and above	3 (12.5)	5 (11.4)	4 (11.4)	23 (14.4)	35 (13.3)
Total	24 (100.0)	44 (100.0)	35 (100.0)	160 (100.0)	263 (100.0)


A statistically significant relationship was found between the number of years in clinical practice and familiarity with national or international antibiotic guidelines (x^2^ = 11.2, P = <0.05).

There was no statistically significant difference in the rate of antibiotic prescription according to sex (P >0.05).

## DISCUSSION

This study examined Turkish dentists’ approaches to antibiotic use in oral surgery, with a particular emphasis on allergy and hypersensitivity management. The results indicate low adherence to guidelines, frequent empirical prescribing, and inadequate allergy management, all of which pose risks for patient safety and AMR. Empirical prescribing predominated, with amoxicillin-clavulanic acid being the most frequently chosen antibiotic, and prophylactic use was reported more often than therapeutic use. Dentists involved in academia showed better adherence to guidelines, likely due to greater access to updated information. However, inconsistencies in practice persist and are often linked to outdated knowledge, excessive caution, or legal concerns. Although Turkey lacks unified dental-specific guidelines, national resources such as the Rational Drug Use National Action Plan and the Turkish Medicines and Medical Devices Agency (TİTCK) provide some direction.^23,24^ These should be harmonised with global frameworks, including the WHO Global Action Plan on AMR, to align with international best practices. While our study used the term ‘national guideline’ to denote existing national resources, no formal dental-specific guideline is available. In contrast, international protocols, such as the ADA’s 2019 guideline11 and the ESE’s 2018 position statement,^18^ offer more structured recommendations. The gap between policy and practice highlights the need for improved dissemination of both national and international guidelines and more frequent training for dental professionals.

To contextualise our results, comparisons were made with international studies. In Shiraz, Iran, more than 40% of dentists reported prescribing antibiotics for conditions where they were not indicated, such as localised fluctuant swellings, a pattern comparable to the inappropriate prescription rates observed in our study.^26^ In Jordan, 56.9% of dentists were unaware of national AMR action plans, suggesting that a lack of awareness and education may drive irrational prescribing.^2^ Similarly, a nationwide study in Spain showed that although awareness of AMR was high, half of the dentists prescribed antibiotics inappropriately in over 28.6% of clinical situations, influenced by knowledge gaps, fear, and complacency.^17^ These findings underscore the complexity of translating awareness into evidence-based prescribing, a challenge also reflected in our results.

To contextualise our results, comparisons were made with international studies. In Shiraz, Iran, more than 40% of dentists reported prescribing antibiotics for conditions where they were not indicated, such as localised fluctuant swellings, a pattern comparable to the inappropriate prescription rates observed in our study.^26^ In Jordan, 56.9% of dentists are unaware of national AMR action plans, suggesting that a lack of awareness and education may drive irrational prescribing.^2^ Similarly, a nationwide study in Spain revealed that although awareness of AMR was high, half of the dentists prescribed antibiotics inappropriately in more than 28.6% of clinical situations, influenced by knowledge gaps, fear, and complacency.^17^ These findings underscore the complexity of translating awareness into evidence-based prescribing, a challenge also reflected in our results.

A Turkish paediatric dentistry study revealed that amoxicillin-clavulanic acid was the most commonly prescribed antibiotic, with clindamycin used for penicillin-allergic patients. Treatment typically lasts 5–7 days, and guideline adherence was 50.9% for therapeutic use and 71% for prophylaxis – both of which are higher than the 33.1% reported in our study.^7^

A Turkish cross-sectional study of dental professionals reported frequent systemic antibiotic use for periodontal diseases – particularly acute necrotising ulcerative gingivitis, aggressive periodontitis, and diabetes-associated periodontitis – largely because of reliance on clinical experience rather than formal training.^6^ Similarly, our study revealed substantial antibiotic use for periodontal conditions, but only 33.1% of participants reported adhering to any guideline, suggesting that limited guideline use contributes to inappropriate prescribing. These patterns underscore the need for targeted interventions, such as electronic prescribing alerts, structured patient counselling, and the integration of guideline reminders into routine practice.

An audit-based US study of more than 88,000 dental visits revealed that only 17.5% of antibiotic use was guideline-appropriate, with a mean duration of 8 days – similar to the extended courses reported in our cohort but contrasted with our participants’ stronger preference for narrow-spectrum agents such as amoxicillin.^20^ A global systematic review further revealed that approximately 70% of dental antibiotic prescriptions are inappropriate and often influenced by fear, complacency, or outdated knowledge,^25^ trends also evident among early-career clinicians in our study.

A Moroccan cross-sectional survey reported extremely high prescribing rates – over 91% for ulcerative necrotising gingivitis and aggressive periodontitis, and nearly 98% for periodontal abscesses.^10^ While both studies document the frequent use of antibiotics for periodontal conditions, our findings add that only 33.1% of clinicians follow formal prescribing protocols, suggesting that poor guideline adherence compounds a problem in our population.

A European review reported that only 18% of patients were routinely informed about antibiotic side effects,^10^ suggesting that dentists in Turkey may perform moderately better in this area. In our study, only 12% of the respondents prescribed prophylactic antibiotics for healthy third-molar extractions. In contrast, 84% of Italian implant dentists routinely prescribed prophylaxis around implant procedures, although only 17% followed the recommendation for a single preoperative dose,^16^ indicating substantially greater use in the Italian cohort.

In this study, 72.6% of the dentists rated their knowledge of AMR as ‘good’ or ‘very good’, whereas 27.4% considered it moderate or low. A pan-European survey reported that 85% recognised AMR as a serious issue, but only 45% felt confident in adjusting their prescribing accordingly.^25^ Thus, while awareness appears comparable, translation into practice seems weaker in our cohort.

The similarities between our findings and those from Europe, the Middle East, and North Africa indicate that empirical prescribing, limited guideline adherence, and inadequate allergy management are widespread challenges that are not unique to Türkiye. This highlights the need for harmonised clinical protocols, wider dissemination of international guidelines, and cross-border continuing education programmes. Our study, therefore, contributes to the growing body of evidence that can inform both regional and global dental policies.

From a clinical perspective, prescribing amoxicillin aligns with appropriate antibiotic use in oral surgery; however, extending treatment beyond 7 days, as reported by 62.7% of respondents, increases the risks of resistance, side effects, and costs. Only 35.4% of dentists consistently informed patients about potential side effects, underscoring gaps in patient communication. Allergy testing and documentation were also limited, highlighting the need for better allergy management. Decision-support tools, electronic allergy alerts, and standardised counselling scripts could enhance both the safety and appropriateness of prescriptions.

This study has notable strengths, including a high response rate and questions directly addressing clinical issues such as allergies and sensitivities. Nonetheless, several limitations should be acknowledged. Because the study was conducted only in Türkiye, the findings may not be generalisable to other countries. Selection bias is possible, as dentists with a greater interest in the topic may have been more likely to participate, and recall bias may have influenced self-reported prescribing habits. Finally, given its cross-sectional design, the study cannot establish causality.

Future research should expand to multiple regions and incorporate qualitative methods, such as interviews, to better understand the drivers of prescribing behaviours. Broader geographic sampling would enhance generalisability, whereas analyses such as audits of actual prescriptions could provide stronger evidence. From a policy perspective, targeted training on antibiotic use – particularly allergy checks and treatment durations – should be integrated into continuing education programmes, and guideline reminders should be embedded into dental record systems. Comparative evaluations of dental education in Turkey and other countries, especially in Europe, may also yield insights for promoting rational antibiotic use. Overall, reducing empirical prescribing, strengthening allergy management, and improving adherence to evidence-based protocols should be prioritised to improve patient safety and strengthen global antimicrobial stewardship.

## CONCLUSION

This study demonstrated that dentists in Turkey frequently rely on empirical antibiotic prescribing during oral surgical procedures, with limited adherence to available clinical guidelines and insufficient attention to allergy management. Although awareness of AMR is relatively high, translation into consistent evidence-based practice remains inadequate. These findings highlight the urgent need for clearer national protocols, the integration of antibiotic stewardship principles into dental education, and improved clinical training on allergy recognition and management. Strengthening guideline-based prescribing and preventive approaches is essential to ensure patient safety and to support global efforts against AMR.

### Acknowledgement

The authors have no acknowledgements to declare.

#### Conflict of interest

The authors declare that they have no conflicts of interest related to the publication of this study.
